# Genetic Algorithm model and data files to assess JONSWAP spectra coefficients: MATLAB code

**DOI:** 10.1016/j.dib.2020.106196

**Published:** 2020-08-19

**Authors:** Juan Gabriel Rueda-Bayona, Andrés Guzmán

**Affiliations:** a Universidad Militar Nueva Granada. Engineering Faculty. Civil Engineering. Water and Energy (AyE) Research Group. Bogotá: Carrera 11 No.101- 80; b Universidad del Norte. Research Group for Structures and Geotechnics (GIEG); Institute for Sustainable Development (IDS). Department of Civil and Environmental Engineering. Km 5 via Puerto Colombia, Bloque K, 8-33K, Barranquilla, Colombia. Postal code: 081007

**Keywords:** Genetic Algorithms, JONSWAP, Numerical modeling, Waves, MATLAB

## Abstract

This data article presents the structure of a Genetic Algorithm model written in a MATLAB code for finding the 1D JONSWAP spectra parameters when measured raw spectra is not available. The JONSWAP spectra is widely used in Coastal, Offshore, and Ocean Engineering for determining wave parameters for structure designing and numerical modeling. However, finding proper spectra parameters may be difficult because of the limitations of parameterized equations to do so and the high non-linear relation between the alpha and gamma coefficients. This GA model can find the alpha and gamma parameters for specific locations considering sea-state's evolution and water-depth transitions. The application of the GA model of this data article is shown in Rueda-Bayona et al. [1].

Specifications Table

**Table:** 

Subject	Ocean engineering
Specific subject area	Wave spectra modeling.
Type of data	Raw
How data were acquired	Coding routines in MATLAB
Data format	MATLAB code, Excel spreadsheet
Parameters for data collection	MATLAB codes were processed in an Intel i7-6820HQ CPU @2.70 GHz, 4 processors, RAM of 16 Gb, PC running Windows (x64). The code was tuned, calibrated and validated through R^2^ correlations, DOE-ANOVA analysis, probability analysis, time-series analysis. The efficiency and robustness of the model were assessed through a population size test for setting the optimum number of chromosomes.
Description of data collection	The MATLAB code was configured as a function and written in American Standard Code for Information Interchange (ASCII) format. Also, an Excel spreadsheet provided as an example input file.
Data source location	Institution: Water and Energy Research Group of Universidad Militar Nueva Granada. City/Town/Region: Bogotá Country: Colombia Latitude and longitude: 4.683165° N 74.041793° W
Data accessibility	Raw data with the article
Related research article	J.G. Rueda-Bayona, A. Guzmán, R. Silva, Genetic algorithms to determine JONSWAP spectra parameters, Ocean Dynamics. 70 (2020) 561–571. https://doi.org/10.1007/s10236-019-01341-8 [1]

Value of the Data

■ The MATLAB code offers a Genetic Algorithm (GA) model to solve the JONSWAP spectra parameters (alpha and gamma) for specific locations, sea-states, and water-depth transitions when *in situ* raw spectra is not available.
■ The user specifies the number of chromosomes and alphas for the mutation that guides to a suitable model solution.
■ The MATLAB tool includes a validation of the model through R^2^ correlations, a p-value of statistical significance, warning messages, and plots for inspection.
■ The MATLAB function generates two ASCII files in txt extension, which gather the identified alpha and gamma parameters from wave parameters (Hs, Tp), that may be used for numerical modeling and offshore structure designing [2].

## 1 Data Description

The MATLAB code gathers the instructions for solving the GA model developed by Rueda-Bayona et al. [1] when wave raw spectra are not available. The MATLAB code is compressed in a folder named JONSWAP_GA.rar along with input in Excel format designated as WAVES.xlsx. The Excel input file gathers hourly significant wave heights and associated wave peak periods of an extreme sea-state generated by Ivan Hurricane during September 2004 in an offshore location of the Colombian Caribbean Sea (La Guajira); details of processing and specific location of the input wave data may be consulted in Rueda-Bayona et al. [2], and Rueda-Bayona J.G [3].

**Fig. 1:**
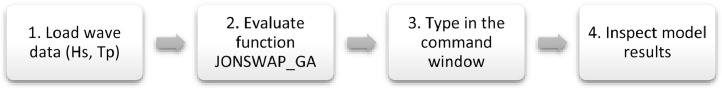
Flow diagram for applying the MATLAB code of the GA model.

**Fig. 2:**
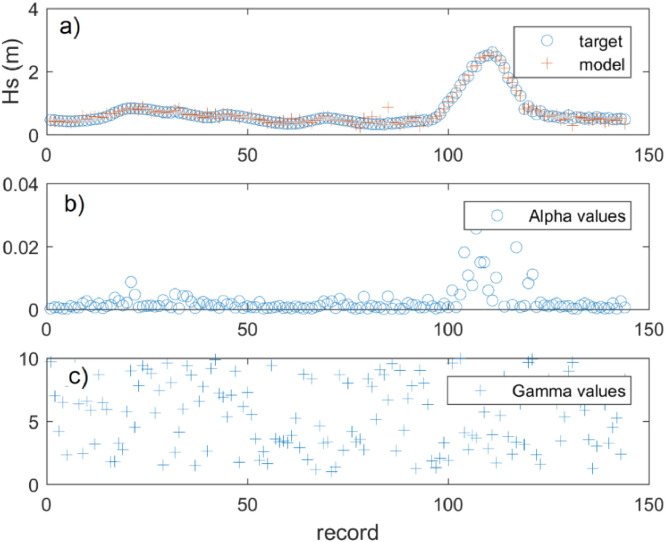
GA model example results a) comparison between input (target) and modeled Hs record, b) generated alpha coefficient for the associated Hs and Tp, c) generated gamma coefficient for the associated Hs and Tp.

The code is written as a MATLAB function (JONSWAP_GA) with internal descriptions to ease the application and understanding of the GA model. The main procedures of the code processing are shown in Fig. 1. Also, the statistical results are printed in the command window and a multiplot (Fig. 2).

## 2 Experimental design, materials, and methods

The theoretical basis of the 1D JONSWAP spectra and the Heuristic procedure of the GA model are described in Rueda-Bayona et al. [1,4], where calibration and validation of the model were performed using *in situ* raw wave spectra. Rueda-Bayona et al. [5] assessed and validated the GA model when *in situ* raw data is not available. The GA model showed its capability for finding suitable alpha and gamma parameters of the JONSWAP spectra for normal and extreme sea events and evidenced the limitations of popular parameterized equations finding the spectra parameters [6].

1 Load wave data (Hs, Tp): In this stage, the user must load the Significant wave height (Hs) and Peak wave period (Tp) data. Both inputs may be scalar or column vector and must have the same element size without NaN nor outlier values.
2 Evaluate function JONSWAP_GA: the user should evaluate the function as JONSWAP_GA(Hs,Tp). The next code lines may be considered as a reference to assess the function mentioned above:

DATA=xlsread('WAVES.xlsx');

Hsig=DATA(:,2); Tp=DATA(:,3);

JONSWAP_GA(Hsig,Tp)

1 Type in the command window: type the number of chromosomes and the number of alpha for mutation when the GA model asks for in the MATLAB command window. For the mutation process, it is recommended a chromosomes group ranging from 50 to 500, and an alpha value of 100. However, the input data may be changed by the user afterward.
2 Inspect model results: the user may check the validation results (R^2^, p-value) and the plotted time-series (Fig. 2).

## Declaration of Competing Interest

The authors declare that they have no known competing financial interests or personal relationships that could have appeared to influence the work reported in this paper.

## Appendix Supplementary materials

Image, application 1

Image, application 2

Image, application 3
