# Supplementary material for: Genetic Algorithm model and data files to assess JONSWAP spectra coefficients: MATLAB code
Source: Data Brief. 2020 Aug 19;32:106196. doi: 10.1016/j.dib.2020.106196 (PMC7452513; doi:10.1016/j.dib.2020.106196)
Supplement: Supplementary file 3 [file mmc3.docx]

CRediT author statement

Juan Gabriel Rueda-Bayona, Ph.D: Writing - Original Draft, Conceptualization, Methodology, Software, Validation, Formal analysis

Andrés Guzman, Ph.D: Writing - Original Draft, Validation.
